# Combined glue embolization and excision for the treatment of venous malformations

**DOI:** 10.1186/s42155-018-0028-y

**Published:** 2018-10-25

**Authors:** Rush H. Chewning, Eric J. Monroe, Antoinette Lindberg, Kevin S. H. Koo, Basavaraj V. Ghodke, Kenneth W. Gow, Patrick J. Javid, Thomas M. Jinguji, Jonathan A. Perkins, Giridhar M. Shivaram

**Affiliations:** 1 Department of Radiology, Section of Interventional Radiology, Seattle Children’s Hospital and University of Washington School of Medicine, Seattle, Washington USA; 2 Department of Orthopedics and Sports Medicine, Seattle Children’s Hospital and University of Washington School of Medicine, Seattle, Washington USA; 3 Department of Surgery, Division of General and Thoracic Surgery, Seattle Children’s Hospital and University of Washington School of Medicine, Seattle, Washington USA; 4 Department of Surgery, Division of Otolaryngology, Seattle Children’s Hospital and University of Washington School of Medicine, Seattle, Washington USA; 5 Department of Radiology, Seattle Children’s Hospital, 4800 Sand Point Way NE, MA.7.220, PO Box 5371, Seattle, Washington USA

## Abstract

**Background:**

The purpose of this study was to evaluate safety, technical success, and clinical outcomes of treatment for venous malformations using *n*-BCA glue embolization immediately prior to excision. Sixty three patients (22 male, 41 female; mean age 12 years (range 1–25)) who underwent 70 procedures for extremity and trunk venous malformations were reviewed. Indications for treatment included pain (100%), swelling (22%), and diminished range of motion (16%). Thirty seven patients (59%) had undergone prior stand-alone interventional or surgical treatment but were persistently symptomatic. Safety, technical and clinical success were retrospectively assessed.

**Results:**

Embolization was technically successful in 100% of patients. Mean lesion size was 3.0 × 2.9 × 5.7 cm. Three patients (5%) underwent planned, second stage procedures for lesions intentionally not treated at the first procedure. Four patients (6%) underwent an unplanned, second stage procedure for residual disease after the primary operation. Mean and median follow-up duration were 18 and 17 months, respectively (range 3 to 35 months). Symptomatic improvement was achieved in 58 patients (92%), of whom 41 (65%) reported complete elimination of pain. There were no recognized instances of nontarget embolization or other complications of the interventional procedure. One patient required additional surgery for wound dehiscence and one patient developed an abscess requiring incision and drainage. Minor surgical complications included surgical site skin infections (*n* = 5) and numbness (*n* = 1). Mean and median surgical blood loss volumes were 131 mL and 10 mL, respectively. One patient required perioperative blood transfusion.

**Conclusions:**

Extremity and truncal venous malformations can be safely and effectively treated in a single-stage fashion using glue embolization immediately preceding excision.

## Background

Venous malformations (VMs) represent the most common type of congenital vascular anomaly, with a prevalence of around 1% (Cahill & Nijs, 2011; Puig et al., 2005). These are structural, non-neoplastic malformations of veins characterized by dilated channels with a single layer of endothelium and a discontinuous smooth muscle covering (Boon et al., 2004) without normal connections to the systemic venous network (Mulliken & Glowacki, 1982). VMs are caused by germline or postzygotic somatic mutations affecting individual cells that locally disturb normal vascular development, either focally or diffusely, with lesions often infiltrating multiple tissue planes (Boscolo et al., 2015; Brouillard & Vikkula, 2007; Limaye et al., 2009; Limaye et al., 2015). Approximately 40% occur in the head and neck, 40% in the extremities, and 20% in the trunk (Cahill & Nijs, 2011).

Clinically, VMs are typically soft and compressible but can become firm if intralesional thrombus develops; they commonly enlarge with Valsalva maneuver (Burrows & Mason, 2004). VMs are present at birth but enlarge as the child grows, usually in proportion to the child’s growth with more accelerated growth seen during puberty. Thus, patients often become symptomatic later in childhood. The majority of patients present with pain and swelling as chief complaints, with bleeding being less common (Burrows & Mason, 2004). When the VM involves a joint or limb, limited range of motion (ROM) and gait disturbance may also occur.

Conservative treatment options, including non-steroidal anti-inflammatory agents and compression garments, may be prescribed when symptoms are mild. However, as symptoms worsen, invasive therapy may be required. While sclerotherapy is the mainstay of invasive treatment and can provide symptomatic relief, multiple treatments are often necessary to achieve a durable response (Burrows & Mason, 2004; Alomari & Dubois, 2011; Bowman et al., 2013). With each treatment session, additional anesthesia, procedural risks, radiation exposure, and costs may be incurred. Stand-alone excision is also feasible, especially for smaller, focal lesions, but potentially presents significant technical challenges ranging from intraoperative bleeding to poor margin delineation for deep or infiltrating venous malformations, often resulting in symptom recurrence (Roh et al., 2012).

Combined percutaneous *n*-BCA glue embolization followed by immediate excision has been previously described as an alternative form of treatment for pediatric head and heck VMs (Tieu et al., 2013). The rationale for this combined approach, performed under a single general anesthetic, is to transform the blood-filled, often ill-defined VM into a hemostatic mass more amenable to excision. For focal lesions, complete excision can be achieved, removing the VM from the body and diminishing the potential for recurrent symptoms. The aim of the present retrospective study was to examine safety, efficacy, and clinical outcomes of this procedure for symptomatic extremity and truncal VMs in a predominantly pediatric patient group.

## Methods

### Patients

Institutional review board approval was obtained for this retrospective study conducted at a large, tertiary-care pediatric hospital. Sixty four consecutive patients who underwent non-head and neck VM glue embolization followed by immediate excision between September 2014 and May 2017 were identified. One patient who underwent glue embolization to control blood loss during planned amputation was excluded.

Seventy procedures were performed in the remaining 63 patients (Table 1). Three patients had discontinuous VMs that were treated with planned, separate procedures. Four patients underwent an unplanned second procedure due to recurrent symptoms with imaging showing residual, unresected VM.

**Table 1:** Patient demographics, treated lesion characteristics, and embolic material volumes

Patient No	Sex	Age (yrs)	Previous treatment	VM Location(s)	Location (SC = subcutaneous, IM = intramuscular, IA = intraarticular, deep non-muscle)	Embolic Volume mL (n-BCA + ethiodized oil)
1	M	6	sclerotherapy	R pretibial/knee	SQ	6
2	M	4	no	R knee	IM, IA	16
		5		R medial ankle	SQ, IM	28
3	M	3	sclerotherapy	R pretibial	SQ, IA	13
		4		R knee	SQ, IA	6
4	M	6	no	R pretibial	SQ	3
5	M	12	surgery	infraumbilical abdominal wall	SQ	6
6	F	23	sclerotherapy	R piriformis/gluteal	IM	7
7	F	9	no	R lateral midfoot	SQ	2
8	F	2	no	R medial midfoot	SQ	2
9	F	2	no	L (a) anterolateral thigh; (b) knee	(a) IM; (b) IA	28
10	F	5	no	R anterolateral thigh, knee	IM, IA	22
11	F	8	surgery, sclerotherapy	R medial hindfoot	IM, SQ	3
12	M	5	no	L forearm	IM	5
13	M	1	no	R posterior calf	SQ	1
		3		R lateral ankle	SQ	2
14	F	13	no	R posterior chest wall	IM	15
15	F	12	no	R subscapular	IM	48
16	F	16	no	L medial calf	SQ	4
17	F	7	sclerotherapy	R hamstring	IM	12
		8		R lateral calf	IM	8
18	M	15	sclerotherapy	L biceps	IM	30
19	M	10	sclerotherapy	L medial calf	IM	3
		11		L medial calf	IM	8
20	F	9	no	L plantar foot, flexor digitorum longus	IM	6
21	M	5	no	R anterior chest	SQ	4
22	F	12	sclerotherapy	R lateral hindfoot	SQ	7
		13		R lateral hindfoot	SQ	4
23	F	17	sclerotherapy, laser	L labia majora	SQ	6
24	F	5	no	R plantar foot	IM	6
25	F	14	sclerotherapy	R anteromedial pretibial	SQ	7
		15		R anterior thigh, suprapatellar	SQ	30
26	F	15	sclerotherapy	L dorsal foot	SQ, IM	3
27	F	17	sclerotherapy	L anterior thigh	IM	8
28	F	14	no	L knee suprapatellar	IA	7
29	F	15	no	L posterior thigh	IM	7
30	F	14	surgery	L back lumbar	SQ, IM	5
31	M	14	surgery	L lateral thigh	IM	30
32	F	18	surgery	L tibialis anterior	IM	1
33	M	22	surgery, sclerotherapy	R plantar foot	IM	12
34	F	14	no	L anteromedial calf	SQ	6
35	M	10	sclerotherapy	L lateral knee	SQ, IA	5
36	F	11	no	R fore/midfoot	SQ, IM	6
37	M	17	sclerotherapy	L vastus medialis	IM	14
38	F	13	sclerotherapy	R pretibial	SQ	11
39	M	5	no	L anterolateral thigh/knee	IM, IA	48
40	M	21	no	L knee	IA	33
41	F	19	surgery, sclerotherapy	(a) L fem neck; (b) patellofemoral	(a) IM; (b) IA	21
42	F	11	no	L medial gastrocnemius/Achilles	IM, deep - intratendinous	2
43	F	7	sclerotherapy	R lateral foot/ankle	SQ	13
44	M	11	sclerotherapy	L lateral thigh (vastus medialis/intermedius), suprapatellar	IM, IA	120
45	F	15	surgery	L plantar foot	IM	20
46	F	15	no	R biceps	IM	9
47	F	14	sclerotherapy	R calf extensor digitorum longus	IM	9
48	F	10	no	R infraspinatus	IM	13
49	F	23	surgery, sclerotherapy	R medial gastrocnemius	IM	11
50	F	15	surgery	L Achilles	deep - intratendinous	3
51	F	7	sclerotherapy	R biceps	IM	28
52	F	18	sclerotherapy	R triceps	IM	13
53	M	16	sclerotherapy	R elbow	SQ	15
54	M	9	no	L elbow	SQ	18
55	F	22	sclerotherapy	R distal thigh/knee	IM, IA	47
56	F	19	surgery	L plantar foot	IM	11
57	F	15	no	L lumbar posterolateral abdominal wall	SQ, IM, deep - retroperitoneal	59
58	M	25	sclerotherapy	L plantar foot	IM	35
59	F	11	surgery	R plantar hindfoot	IM	5
60	M	11	surgery	R (a) anterior thigh; (b) lateral knee; (c) gluteal	SQ	10
61	M	10	surgery	L soleus	IM	7
62	F	14	no	R medial hindfoot	SQ	2
63	F	18	surgery	R upper calf	IM, SQ	3

Symptoms at presentation included pain (*n* = 63; 100%), swelling (14; 22%), limited ROM (10; 16%), gait disturbance (4; 6%), and bleeding (1; 2%). Mean age at treatment was 12 years (range 1–25 years). Twenty-two patients were male (35%) and 41 were female (65%). Locations of VMs included lower extremity (48; 76%), trunk/pelvis (8; 13%), and upper extremity (7; 11%). Prior treatment included sclerotherapy alone (22; 35%), surgery alone (11; 17%), and both surgery and sclerotherapy (4; 6%). Twenty-six patients (40%) had no prior surgery or sclerotherapy. Initially, glue-excision treatment was offered to patients who had refractory symptoms despite repeated treatments with sclerotherapy or excision alone. Later, patients presenting without a history of prior treatment were also offered this procedure as an alternative to sclerotherapy or excision alone. All patients required oral non-steroidal anti-inflammatory medications for symptoms prior to treatment. Demographic information is summarized in Table 1.

The electronic medical record was used to retrospectively review all images and clinical data. Most recent clinical follow-up documentation determined the duration of follow-up.

### Embolization technique

**Fig. 1:**
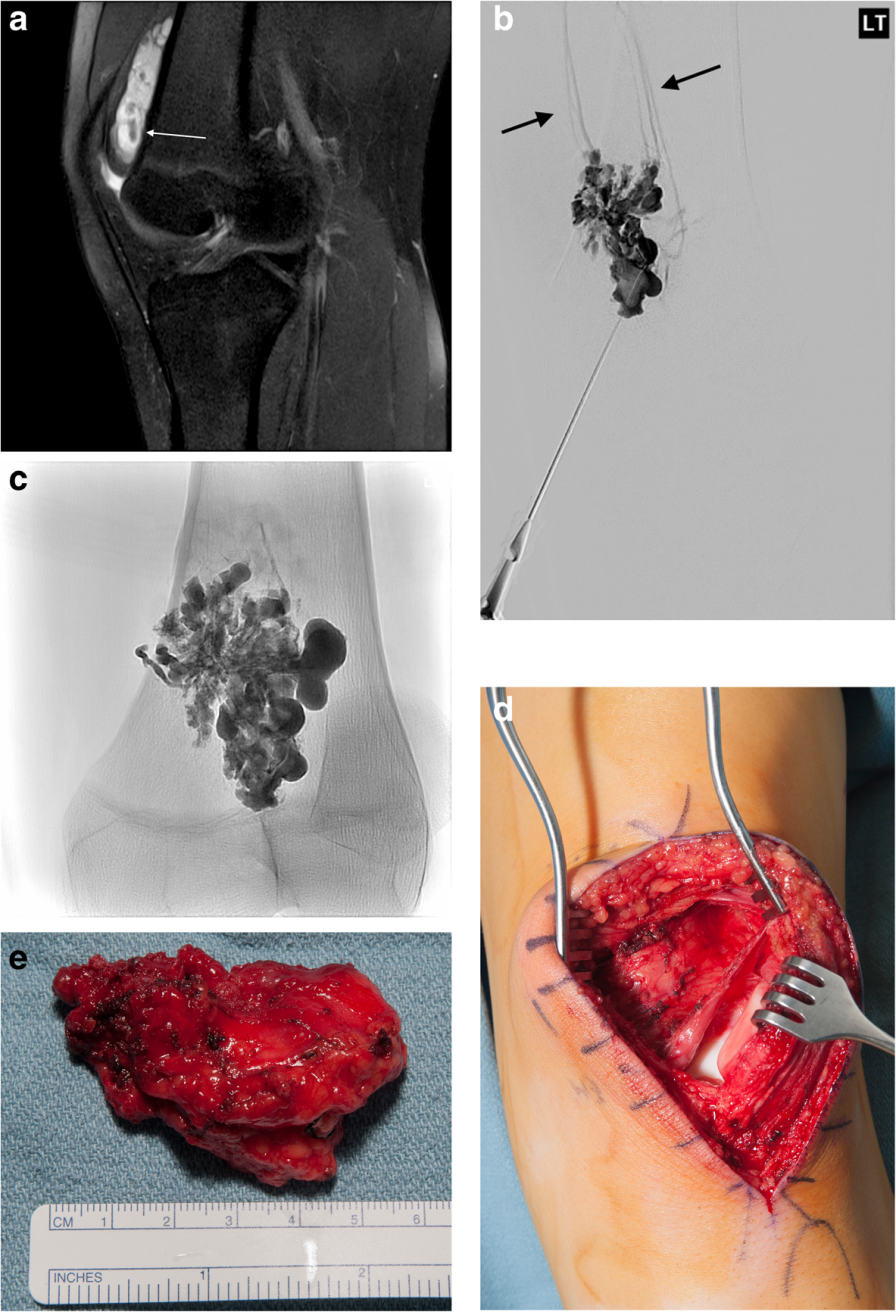
**a** 14-year-old female with chronic left knee pain and swelling initially misdiagnosed and managed as monoarticular juvenile idiopathic arthritis. Sagittal fat saturated proton density-weighted MRI showing hyperintense channels of the intraarticular venous malformation along with hypointense phleboliths (arrow). The lesion measured 3.4 × 0.9 × 4.5 (SI) cm. **b** Frontal projection digital subtraction venography after direct percutaneous access of the venous malformation showing opacification of numerous channels with ascending venous outflow (arrows). **c** *Frontal* completion spot radiograph after delivery of *n-*BCA glue mixture into the venous malformation showing no nontarget egress of glue into draining veins. 7.2 mL of glue-ethiodized oil mixture were delivered. **d** Intraoperative photograph showing excision cavity after en bloc excision of glue-filled venous malformation. **e** Intraoperative photograph showing gross specimen of glue-filled venous malformation, which was resected en bloc

**Fig. 2:**
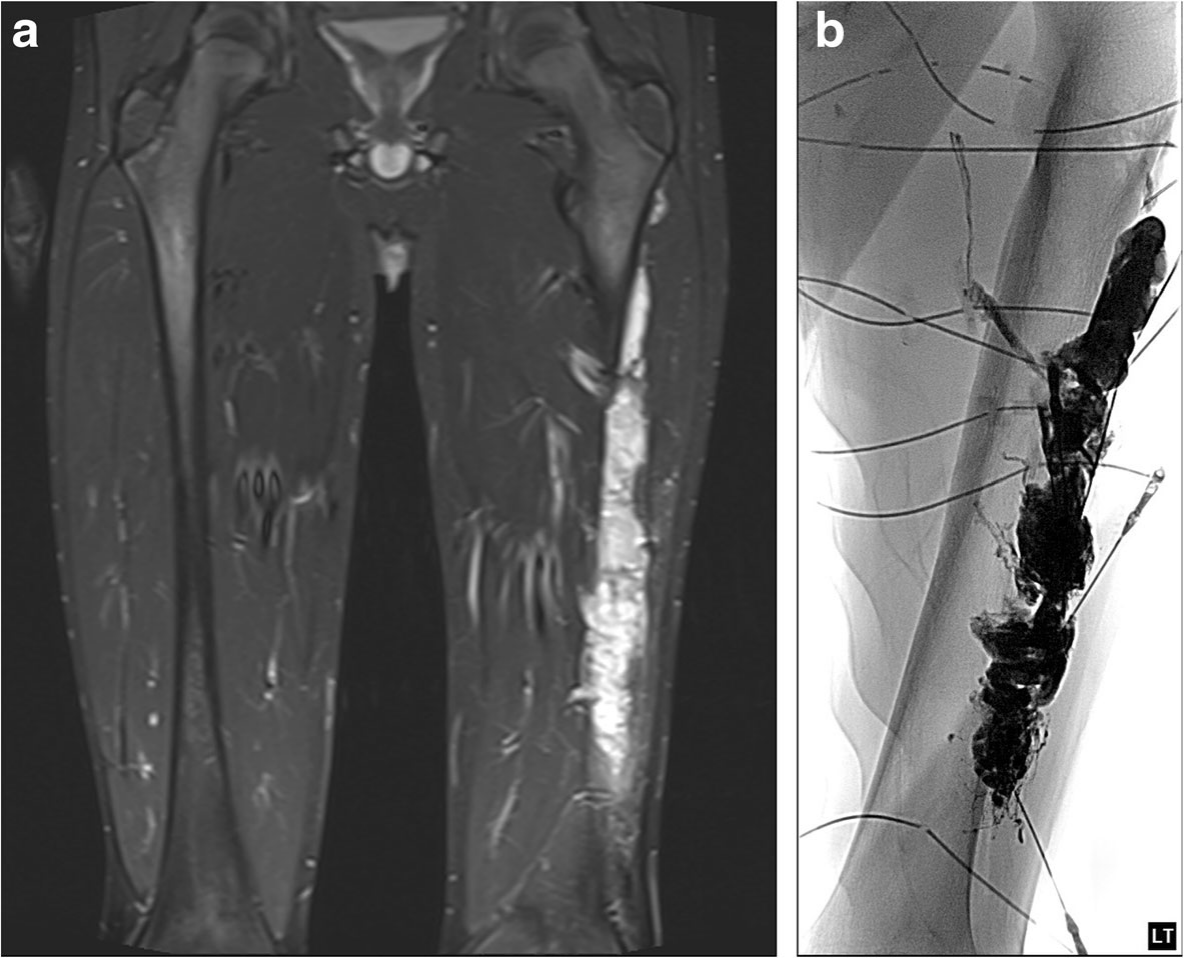
**a** Pre-operative coronal T2 weighted MRI in a 14-year-old male with a large (2.7 × 3.2 × 21.2 cm) intramuscular left vastus lateralis VM. He had undergone 3 prior stand-alone excisions but had persistent symptoms and large residual VM. **b** Post-embolization frontal spot image showing *n*-BCA glue embolization of the lesion, using multiple points of percutaneous access

All patients underwent preoperative consultation in both interventional radiology and orthopedic surgery or general surgery clinics. Pre-operative non-invasive imaging was obtained in all patients to confirm the diagnosis of VM, identify targets suitable for percutaneous access, and to define lesion extent and feasibility of excision. Doppler ultrasound (US) was obtained in all cases and contrast-enhanced magnetic resonance imaging (MRI) (Figs. 1a, 2a) was obtained prior to 67 of the 70 procedures performed. All embolization procedures were performed by one of two board-certified interventional radiologists (E.J.M and G.S.) with 3 and 5 years of experience respectively.

All patients underwent a single general anesthetic, induced in the interventional radiology suite, for both components of the combined procedure. 21-gauge (Cook Medical, Bloomington, IN, USA) or 23-gauge (Beckton Dickinson, Franklin Lakes, NJ, USA) needles were advanced into channels of the VM under continuous US or intermittent fluoroscopic guidance. In most cases, multiple points of access were required. Contrast (Optiray 320, Mallinckrodt Pharmaceuticals: St. Louis MO USA) venography was performed through the needle prior to delivering embolic to delineate angioarchitecture of the VM (Fig. 1b). Rate of washout and location of draining veins were assessed in order to minimize risk of nontarget embolization.

Embolization was then performed using *n*-BCA glue. Trufill glue (Codman Neuro Division - DePuy Synthes: Raynham, MA USA) was used in the first two procedures and subsequently Histoacryl glue (B. Braun Melsungen AG: Melsungen, Germany) for all remaining procedures, as the latter offered approximately 1:100 cost reduction.

The mixture of *n*-BCA glue in ethiodized oil (Lipiodol Ultra-Fluide, Guerbet LLC: Villepinte, France) was prepared on a separate, ion-free table. The ratio of *n*-BCA to ethiodized oil ranged from 1:3 to 1:5 with a mean of 1:4. Fluoroscopy with negative roadmap technique was used to monitor injection of the glue mixture into the VM. Once the lesion was completely filled with the liquid embolic mixture, the needles were removed and hemostasis was achieved with gentle manual compression (Figs. 1c, 2b). Post-procedural cone beam computed tomography was preformed (XperCT, Philips: Eindhoven, the Netherlands) in the majority of cases and 3-dimensionally rendered images were reconstructed to aid in surgical planning. Immediately following glue embolization, patients were transported while still under general anesthesia from the interventional suite to the operating room for VM glue mass excision by a board certified pediatric orthopedic or general surgeon (Fig. 1d-e).

### Definitions and endpoints

Technical success of the embolization procedure was defined as complete embolization of the lesion targeted for operative excision. Technical success of the operative procedure was defined as complete excision of the embolized lesion. When patients were later found to have an unplanned need for a second procedure, this was deemed as technical failure of the operative procedure. Clinical success was defined as symptomatic improvement and the lack of need for additional procedures. Procedural and operative notes were used to assess technical success of the embolization, procedural complications, and estimated blood loss at surgery. Post-procedure clinic notes and follow-up telephone calls were used to assess delayed complications, symptom improvement, and need for additional treatment. All follow-ups included assessment of pain on a numeric rating scale (NRS) from 0 to 10, medication usage, bleeding, and functional limitations. Pre-procedure NRS assessment of pain was not reliably available. ROM assessments were performed by a board-certified pediatric orthopedic surgeon (A.L.).

## Results

### Technical and clinical success

Technical success of preoperative embolization was 100%, whereas technical success of excision was 94%. Mean lesion size was 3.0 ± 1.8 (ML) × 2.9 ± 2.0 (AP) × 5.7 ± 4.7 cm. Mean *n*-BCA volume (not including ethiodized oil) injected was 2.7 mL (range 0.2–20 mL). Mean volume of total embolic mixture (*n-*BCA glue and ethiodized oil) was 14.4 mL (range 1–120 mL).

Mean and median follow-up duration were 18 and 17 months, respectively. Three patients (5%) underwent a planned second stage procedure either for their originally treated lesion or a lesion at a separate anatomic site. Four patients (6%) underwent an unplanned second stage procedure for symptomatic residual disease. After completion of single or double stage glue embolization and excision, symptom improvement was achieved in 58 of 63 patients (92%), of whom 41 (65%) reported complete elimination of pain. One patient received additional surgery (excision of medial head of the gastrocnemius muscle) at an outside hospital for persistent calf pain following glue embolization and excision of the patient’s intramuscular calf VM. This was done despite that post-surgical MRI did not show a residual VM within the muscle and complex regional pain syndrome (CRPS) had already been established as a secondary diagnosis. With inclusion of this patient with the 4 patients who required unplanned, second stage procedures, a total of 5 patients (8%) required unplanned second surgeries. No patients received sclerotherapy or stand-alone surgical treatment following the glue embolization and excision procedure. Fifty-two patients (83%) were no longer using pain medications following the procedure at last follow-up. 45 (71%) patients were able to resume full activities following treatment. Mean and median pain scores at last follow-up on NRS of 1–10 were 1.2 and 0, respectively.

### Complications

There were no instances of recognized large vessel nontarget embolization, deep venous thrombosis, bleeding or other complications of the interventional procedure. One patient required additional surgery for wound dehiscence and one patient developed an abscess requiring incision and drainage. Minor surgical complications included surgical site skin infections (*n* = 5) and foot numbness (*n* = 1). This instance of foot numbness was presumed secondary to surgical injury but could have been from unrecognized microvascular nontarget glue embolization. Mean and median surgical blood loss volumes were 131 mL and 10 mL, respectively (range 0 to 2300 mL). One patient required perioperative blood transfusion.

## Discussion

The use of *n*-BCA for immediate pre-excisional embolization of extremity and truncal VMs was found to be safe and technically feasible in the present study. Clinical success, as defined by symptom improvement and freedom from unplanned reintervention, was 92% according to both measures. 65% of patients treated reported complete elimination of pain. No complications occurred during the embolization procedures; surgical complications were mostly minor with only two patients requiring additional surgery to manage wound complications.

Percutaneous sclerotherapy and stand-alone excision are both well-established techniques for invasive treatment of VMs (Cahill & Nijs, 2011; Burrows & Mason, 2004; Alomari & Dubois, 2011; Bowman et al., 2013). However, these approaches can be limited by the need for multiple treatments due to incomplete treatment in a single session, which may be conceptually explained by incomplete destruction or removal of VM channels. Percutaneous *n*-BCA glue embolization followed by immediate excision has previously been described for pediatric head and neck VMs (Tieu et al., 2013), and in general the use of cyanoacrylates for embolization of vascular malformations has been described for a variety of lesion types (Rosen & Contractor, 2004; Pollak & White, 2001; Lee, 2005). The rationale for the combined approach as described by Tieu, et al. for head and neck VMs was to limit the morbidity of multiple procedures requiring multiple anesthetics in children by attempting complete single-stage removal of VMs (Tieu et al., 2013). Glue polymerization within the VM converts what is otherwise an ill-defined, blood filled lesion into a firm mass that is more easily resected while potentially minimizing operative blood loss or loss of unaffected adjacent tissues. In this way, glue embolization affords the surgeon the ability to palpate the firm VM distinctly from surrounding muscular or other soft tissue, which helps to achieve a more complete excision. Finally, complications of ethanol or detergent sclerotherapy, including skin injury, nontarget embolization, and systemic toxicity are minimized or avoided altogether (Burrows & Mason, 2004; Alomari & Dubois, 2011; Bowman et al., 2013; Lee, 2004; Qiu et al., 2013).

In the current cohort, initial patients offered percutaneous glue embolization followed by immediate excision for extremity and truncal VMs had failed percutaneous sclerotherapy or stand-alone excision. Because of encouraging clinical response in these initial patients compared to prior treatments, the technique was later offered to patients who had not previously undergone any kind of invasive treatment. During the course of this experience, it was appreciated that alteration of the angioarchitecture of the VM by prior sclerotherapy or surgery resulted in greater technical difficulty of both embolization, due to more discontinuity between VM channels compared to untreated lesions, and excision, due to scar tissue formation. In several cases, proximity to nerves was identified during excision, which could explain why some patients had symptoms exacerbated by prior sclerotherapy procedures. Initially, large lesion size was felt to be a contraindication to the procedure because of large embolic volume required and increased complexity of excision. However, over time, larger lesions were treated using this technique, with comparable clinical results to more focal lesions.

Potential limitations of this technique are that it requires a longer single anesthetic than a stand-alone procedure, requires more coordination between departments, and usually requires safe transportation of an intubated patient from one location in the hospital to another.

This report contains several limitations, largely stemming from its retrospective and non-controlled nature as well as heterogeneity of patients’ VMs and prior treatments. Furthermore, typical of early experience with a novel procedure, patient inclusion as well as interventional and surgical technique varied over the study period as the institutional approach to VM treatment evolved and greater technical experience was gained. The study conclusions would be significantly strengthened by standardized patient inclusion and controlled comparison with conservative treatment, sclerotherapy, or stand-alone excision.

## Conclusion

In conclusion, glue embolization followed by immediate excision is technically successful and safe for single-stage treatment of extremity and truncal VMs with good intermediate-term clinical success. Longer-term follow-up will be needed to ascertain maintenance of symptom control and freedom from reintervention.

## Abbreviations

*n*-BCA: *n*-butyl cyanoacrylate
ROM: Range of motion
VM: Venous malformation
